# Solitary pulmonary hyalinising granuloma: a rare cause of pulmonary nodule

**DOI:** 10.1259/bjrcr.20160055

**Published:** 2016-11-26

**Authors:** Muhammad Adnan Saleem, Rahul Bhat, Bidisa Sinha

**Affiliations:** ^1^Respiratory Medicine, George Eliot Hospital, Nuneaton, England; ^2^Respiratory Medicine, University Hospitals Coventry and Warwickshire, Coventry, UK

## Abstract

A pulmonary nodule is a common incidental finding on chest imaging, which includes a wide variety of differential diagnosis. Pulmonary hyalinising granuloma is a rare disease aetiology of pulmonary nodule(s). We report a 74-year-old female who was referred to the respiratory clinic with incidental finding of a solitary pulmonary nodule on chest X-ray. CT confirmed the presence of a 1.2 cm solitary pulmonary nodule in the left upper lobe with no lymphadenopathy. The patient underwent wedge resection, and histopathological examination of the lesion confirmed pulmonary hyalinising granuloma. In most previously reported cases, patients had multiple lesions on chest radiography. Solitary pulmonary lesion is an uncommon presentation of this clinical entity and only a few cases have been reported in the literature.

## Background

Pulmonary hyalinising granuloma (PHG) is a rare condition, presenting more often as multiple pulmonary nodules. It is characterized by fibrosing nodules, consisting of central whorled deposits of lamellar collagen. The nodules are benign in their clinical course but may slowly grow in size. Often, a biopsy is required to establish the diagnosis of PHG. Presentation as a solitary nodule is particularly rare.

## Case presentation

A 74-year-old female was referred to the respiratory clinic with incidental finding of a pulmonary nodule on chest X-ray. She did not have any other respiratory symptoms. She was a non-smoker throughout her life and had no exposure to asbestos or tuberculosis. Her past medical history was significant only for chronic lymphoedema and recent hip replacement.

## Investigations

**Figure 1.**
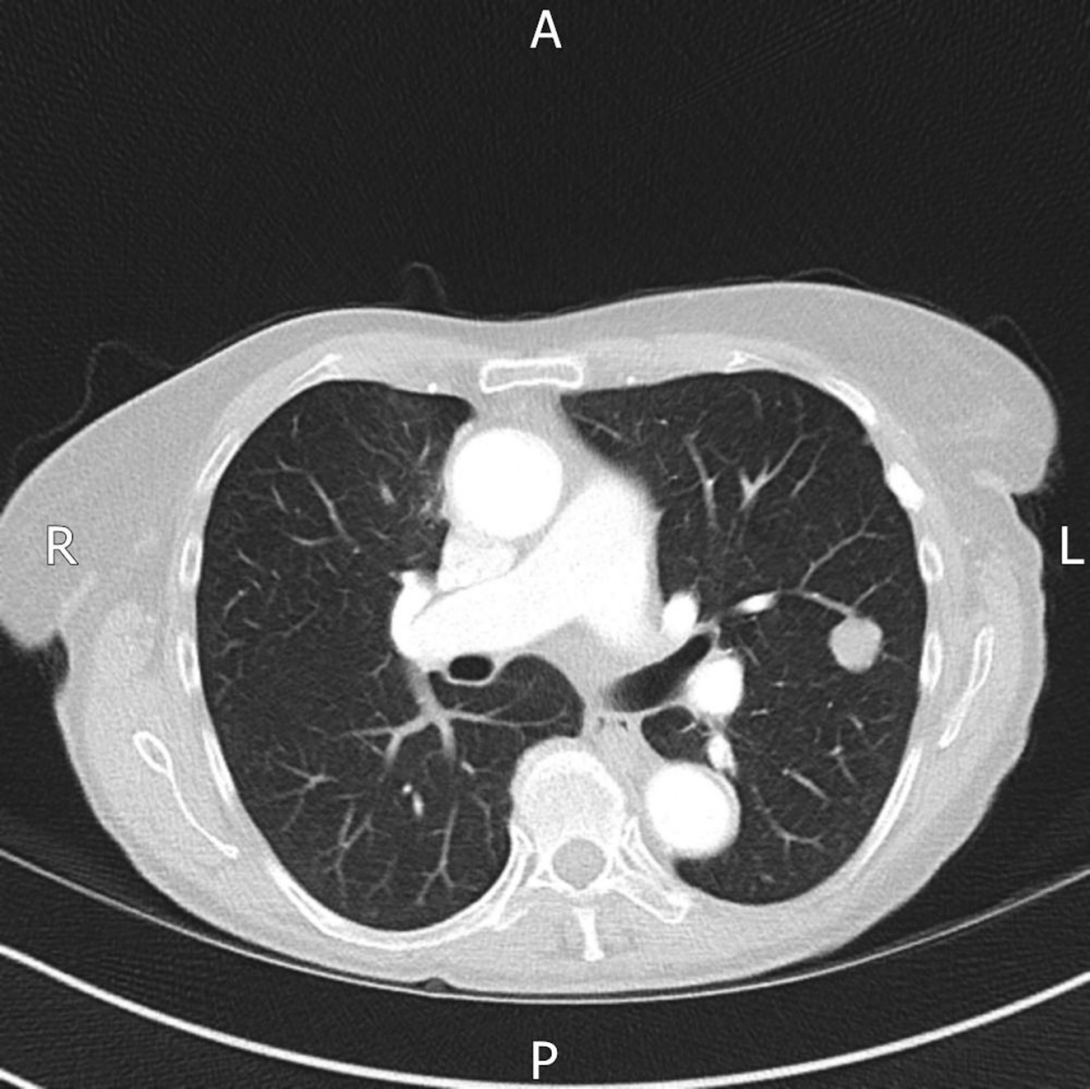
CT scan of thorax shows 1.2 cm pulmonary nodule. No associated mediastinal lymphadenopathy is seen. No other discrete nodules are present.

Subsequently , CT thorax was performed, which confirmed a 1.2 cm pulmonary nodule in the posterior segment of the left upper lobe. There was no cavitation or calcification and no discrete nodules were present in the remaining lung parenchyma. There was no mediastinal or hilar lymphadenopathy (Figure 1).

**Figure 2. Positron emission tomography:**
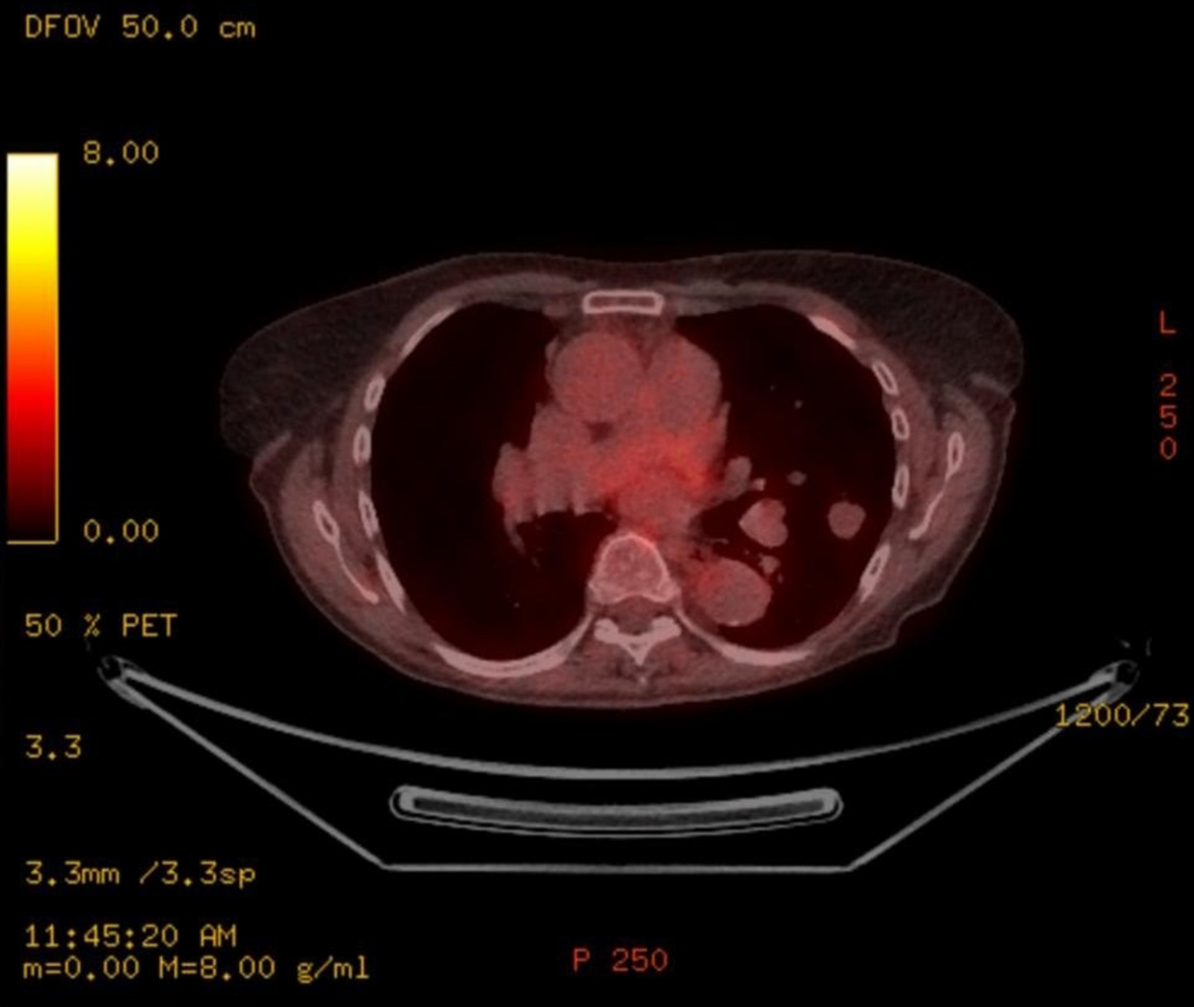
scan shows lobulated pulmonary nodule, not significantly avid to be diagnostic of malignancy.

On positron emission tomography scan, the pulmonary nodule was found to be non-avid (Figure 2). Her pulmonary function tests were normal with a normal gas transfer. She was also investigated for any underlying plasma cell dyscrasias to rule out nodular amyloid deposit: no evidence was found to support it as her serum and urinary electrophoresis was negative with normal immunoglobulin levels. Biopsy of the lesion was not feasible either by bronchoscopy or by CT-guided lung biopsy. The patient underwent wedge resection as she was keen to have a definitive diagnosis and did not want to have serial imaging as part of pulmonary nodule surveillance. Microscopy of the fluid sample from the wedge biopsy showed scanty lymphocytes. No pathogenic microorganisms were found. Mycobacterial and fungal cultures were also negative.

**Figure 3.**
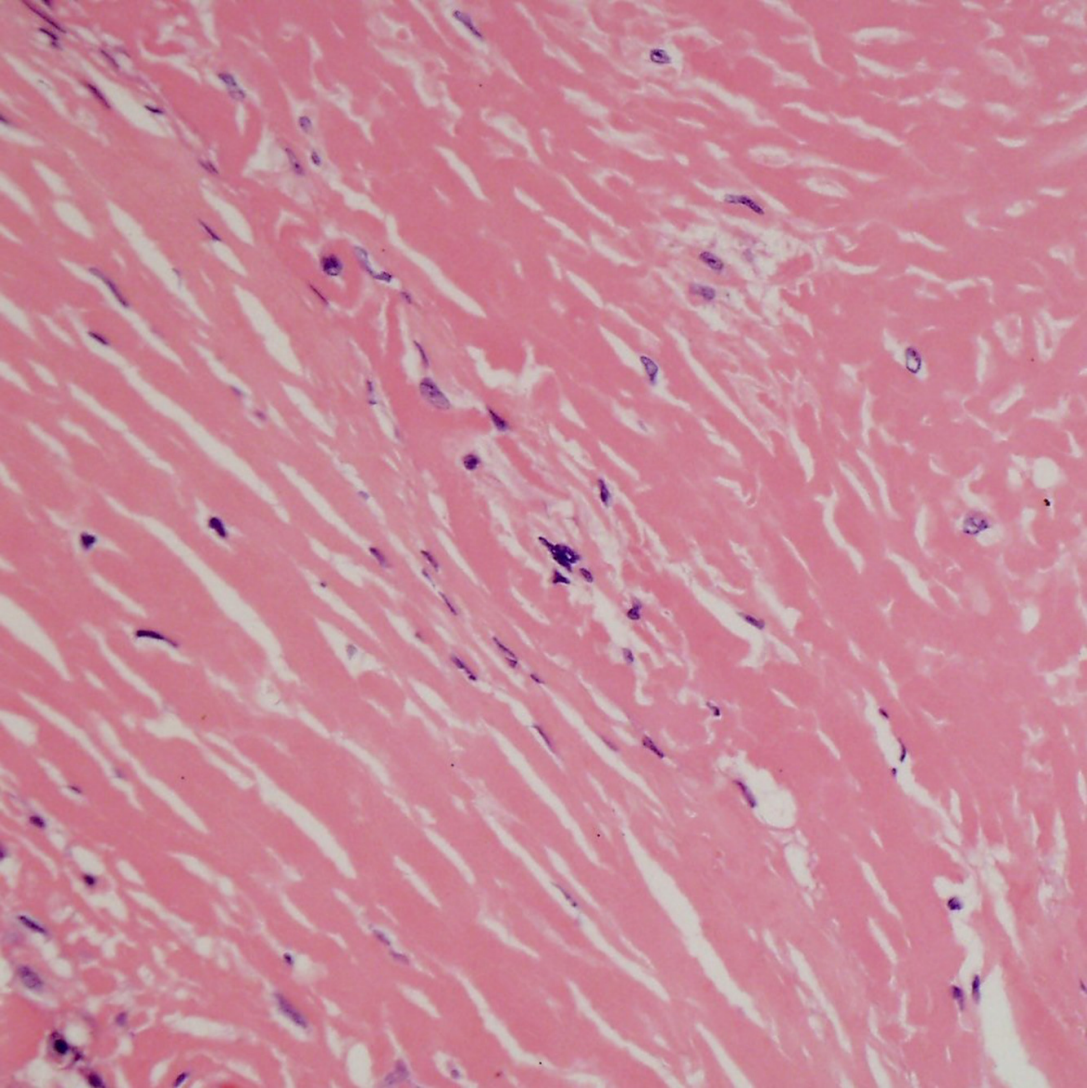
Whorls of lamellar fibrosis.

Histology showed abundant eosinophilic material with scattered giant cell reaction. No necrosis or epithelioid granulomatous inflammation was seen. Lamellar fibrosis was evident (Figure 3).

**Figure 4.**
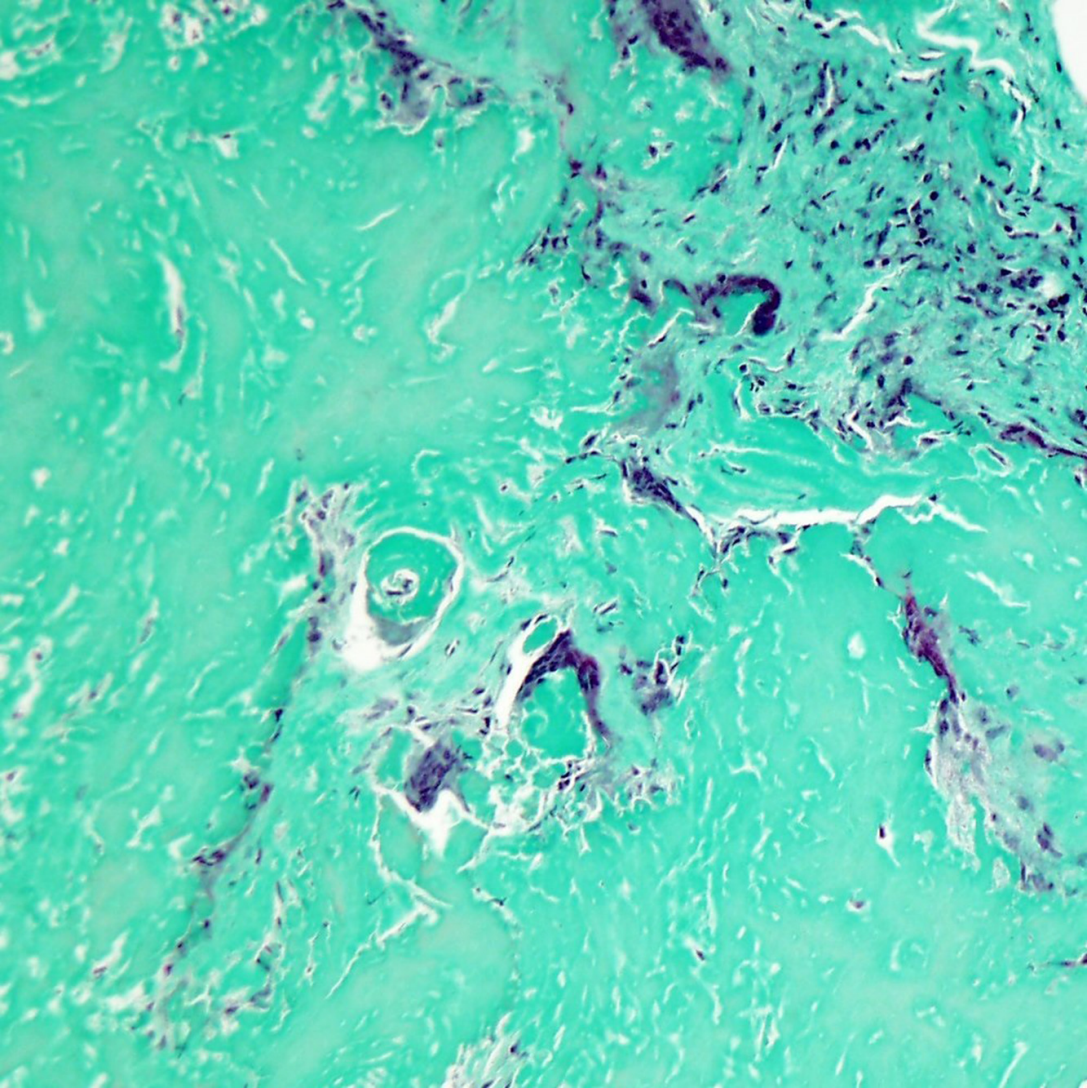
Masson’s stain confirms collagen (green).

**Figure 5.**
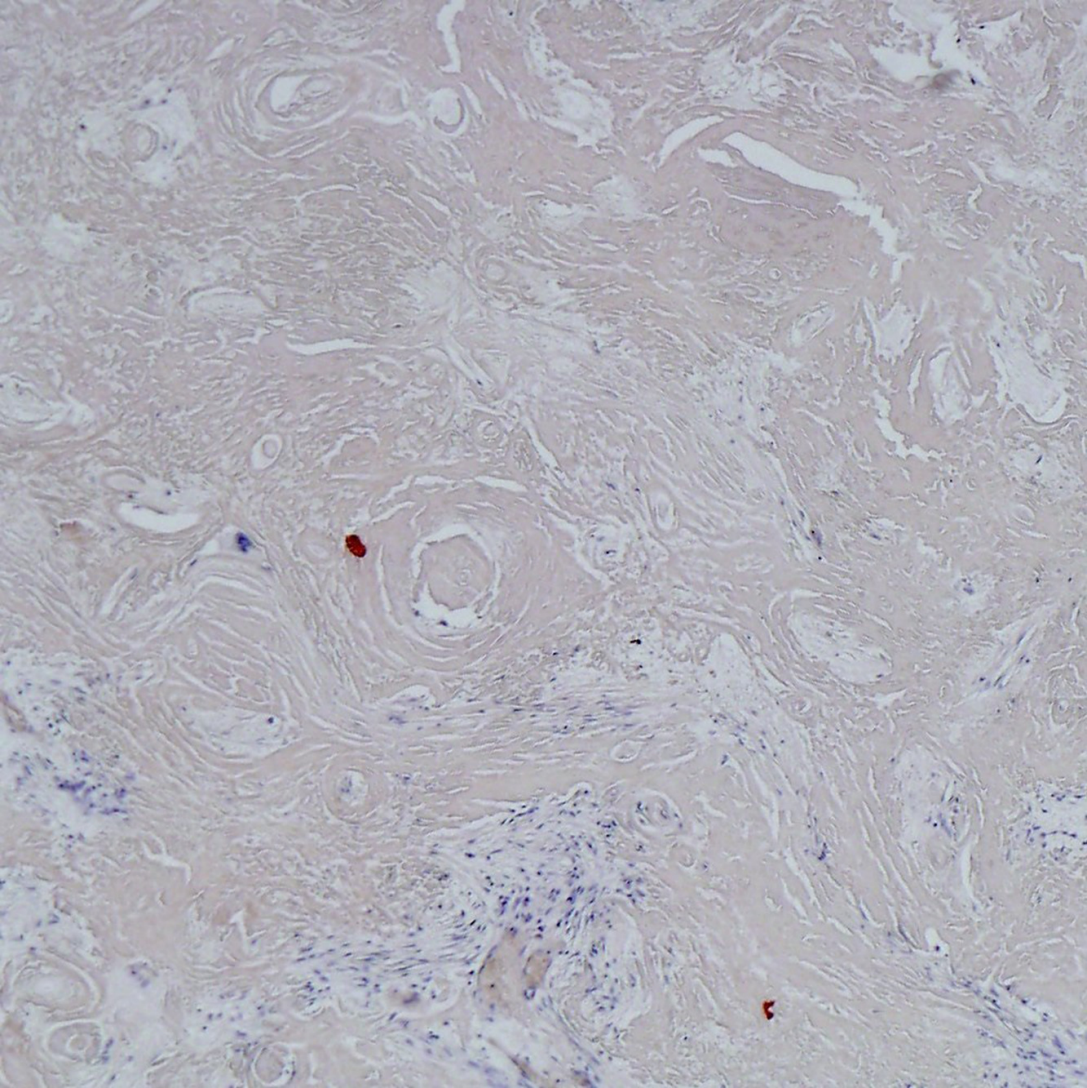
Negative Congo red stain for amyloidosis.

Masson’s stain confirmed hyaline lamellar fibrosis whereas Congo red stain for amyloidosis was negative (Figures 4 and 5). These features are consistent with the diagnosis of PHG.

## Differential diagnosis 

PHG is included in the differential diagnosis of diseases such as infectious granulomatous diseases (tuberculosis, histoplasmosis), non-infectious granulomatous diseases (sarcoidosis, Wegener and plasma cell granuloma), amyloidosis and rheumatoid lung involvement.

## Treatment

The patient had nodule excision through wedge biopsy.

## Outcome and follow-up

The patient is stable after surgery and no complication has been reported on follow-up.

## Discussion

PHG was first described in 1977.^1^ The mean age of presentation is 45, with an age range of 15–77.^1,2^ There is no gender or racial predilection.^2^ It could present with vague symptoms. In 25% of cases it is asymptomatic and is discovered incidentally on routine chest radiographs.

In most cases, PHG presents as multiple, unilateral/bilateral pulmonary nodules with well-defined circumscribed borders. Lesion size ranges from a few millimetres to 15 cm, with an average size of 2 cm.^1,2^ Solitary pulmonary nodules are rare.^3,4^ Usually they grow slowly, or may not grow at all; spontaneous regression has also been reported. Positron emission tomography-CT may or may not reveal increased metabolic activity in PHG lesions, although it is useful in ruling out metastatic lesion.^5^

 The aetiology of PHG remains unclear. It is postulated that it results from abnormally elevated immune response to one of the various infectious or autoimmune processes. Various associations of PHG have been reported in the literature, such as fungal or mycobacterial lung disease, rheumatoid arthritis, lupus-like anticoagulant, sclerosing mediastinitis, sarcoidosis, IgG4-related sclerosing disease, idiopathic thrombocytopenic purpura and lymphoproliferative disorders.^1,2,5–9^ However, our patient did not have any of the above associations, and clinically there was no evidence of any autoimmune disease.

Definitive diagnosis of PHG may require a wide excisional biopsy. Histologically, it is described as a densely fibrotic nodule with patchy chronic inflammation around. The nodules are composed of thick, hyalinized bands of collagen that are arranged in whorls.^10^

The prognosis of PHG is generally good with no known malignant potential. Solitary lesions tend to be stable. Some patients with multiple lesions may show progressive enlargement of nodules and worsening dyspnoea. There are some reports of recurrence after resection. There is no definitive treatment but successful resolutions of the multiple lesions have been reported after administration of glucocorticoids.^11^

## Learning points

1. Sometimes, it is difficult to differentiate radiologically the true nature of a pulmonary nodule and biopsy is required for definite diagnosis.
2. Although solitary nodule PHG is rare, this case report demonstrates one such case and hence should be considered as differential diagnosis.
3. We should actively look for any associated condition in a patient diagnosed with PHG.

## Consent

Written informed consent was obtained from the patient for the publication of this case report,including accompanying images.
